# Supplementary figures and images for: Precision Autism: Genomic Stratification of Disorders Making Up the Broad Spectrum May Demystify Its “Epidemic Rates”
Source: J Pers Med. 2021 Oct 30;11(11):1119. doi: 10.3390/jpm11111119 (PMC8620644; doi:10.3390/jpm11111119)

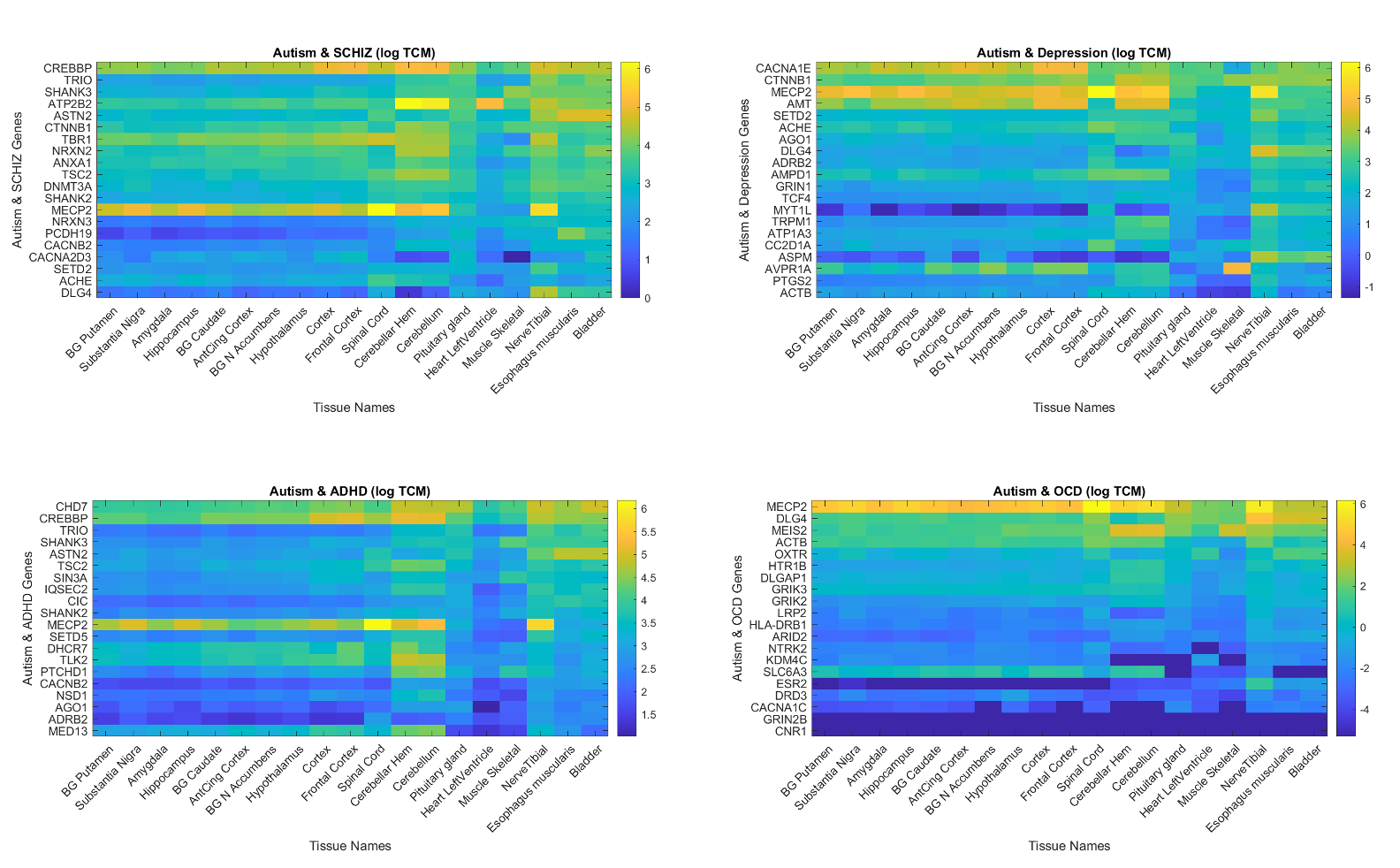

Supplement: Supplementary file 1 [file jpm-11-01119-s001.zip › Supplementary Figure S1.tiff]

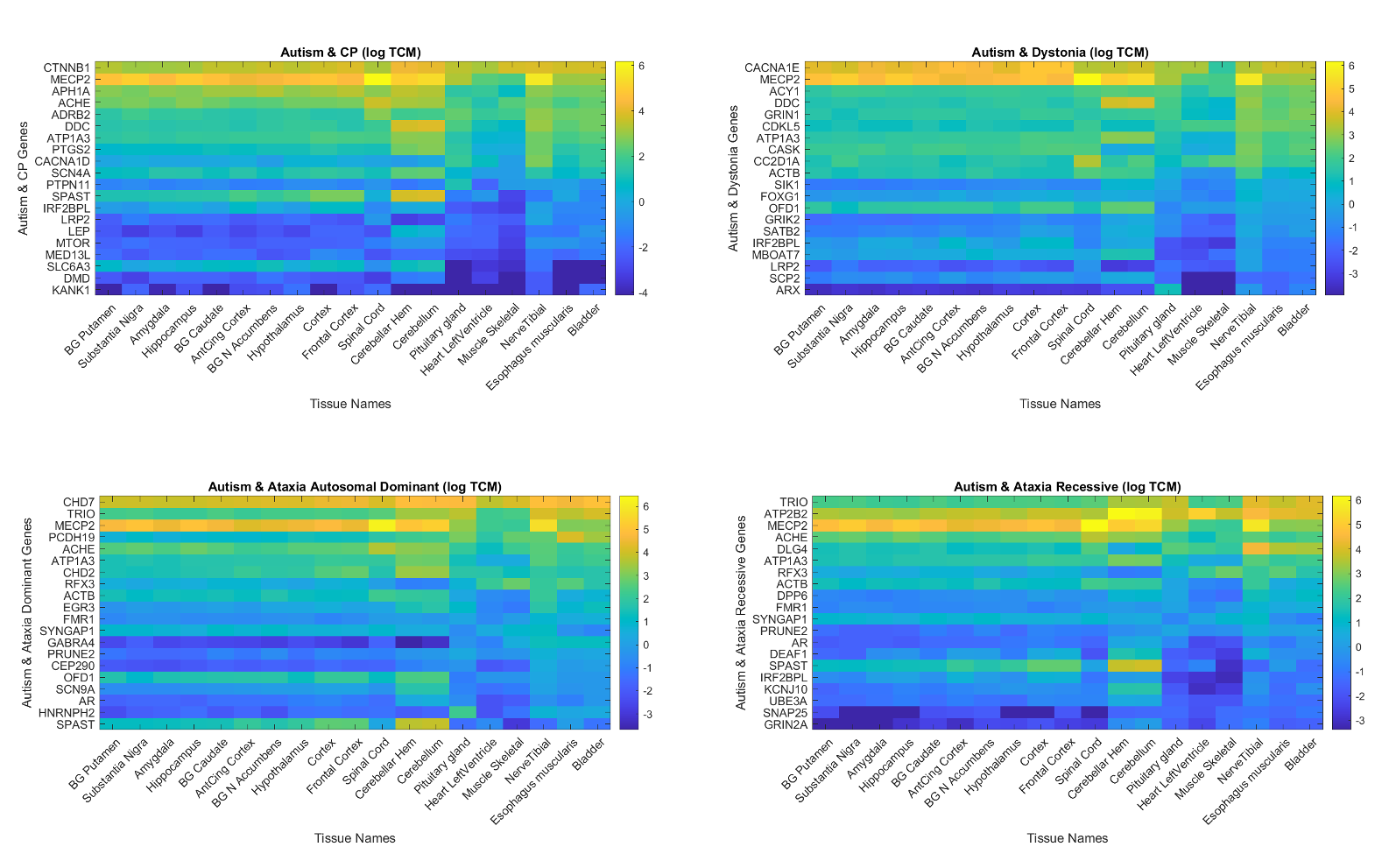

Supplement: Supplementary file 1 [file jpm-11-01119-s001.zip › Supplementary Figure S2.tiff]

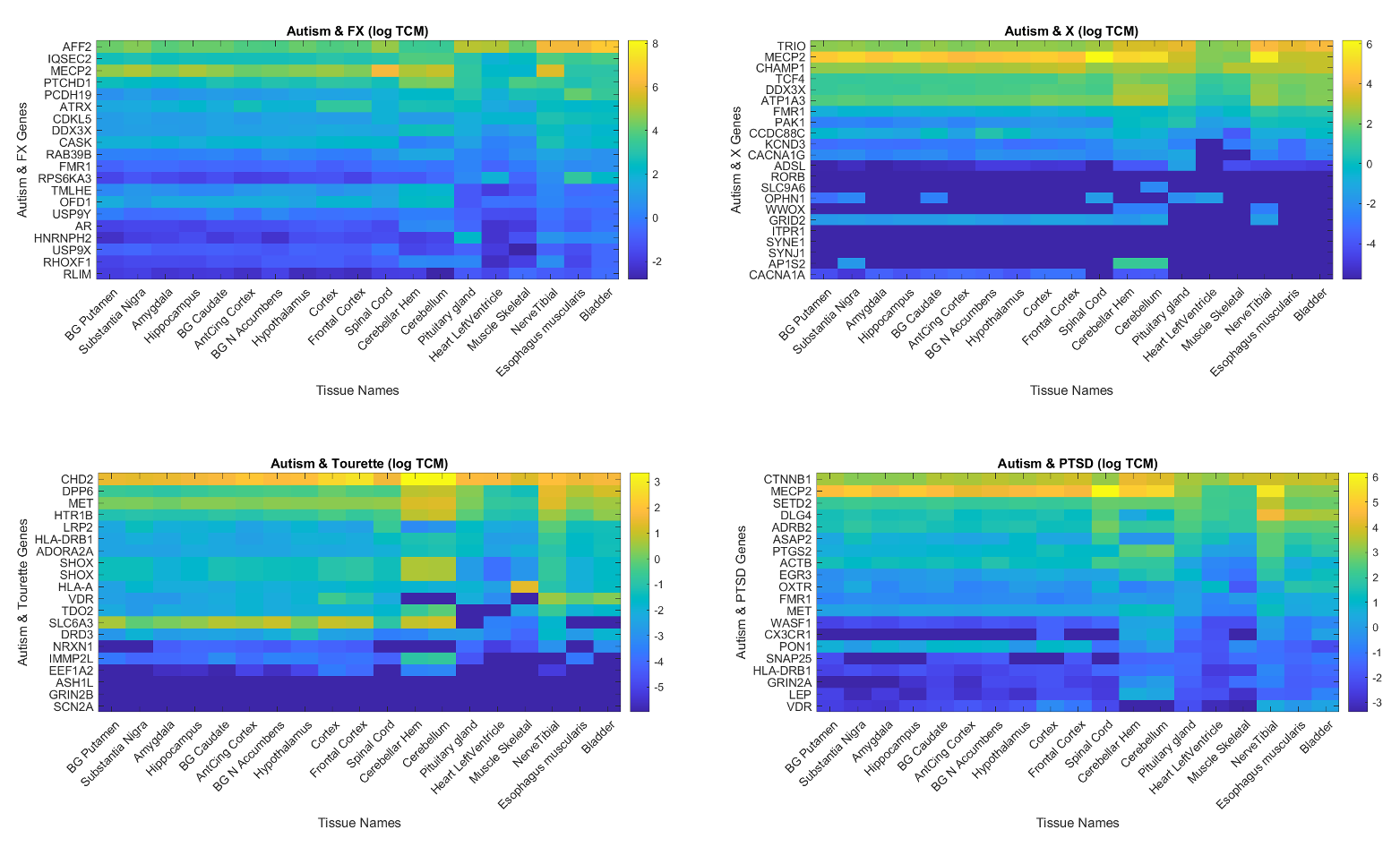

Supplement: Supplementary file 1 [file jpm-11-01119-s001.zip › Supplementary Figure S3.tiff]

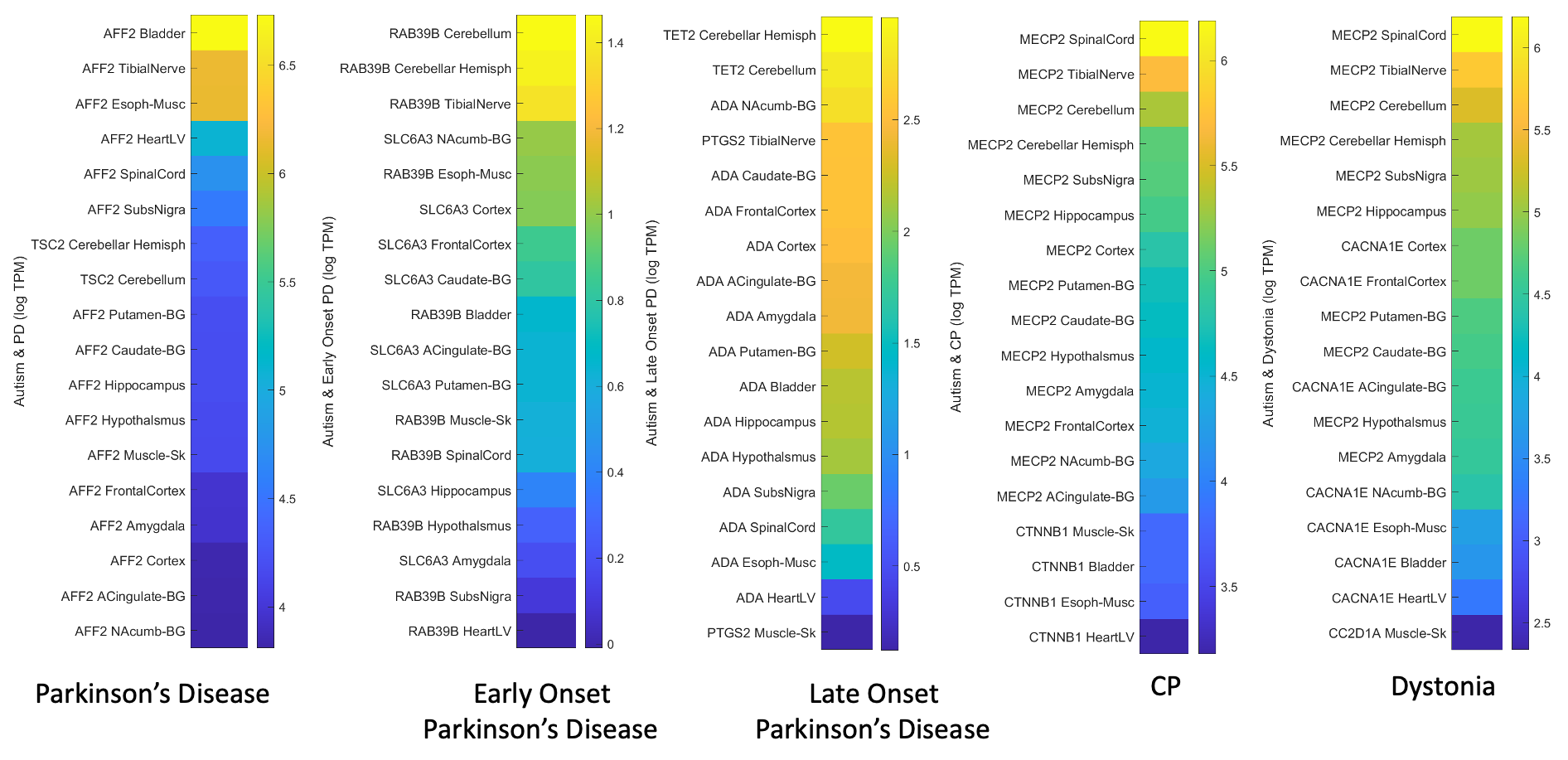

Supplement: Supplementary file 1 [file jpm-11-01119-s001.zip › Supplementary Figure S4.tiff]

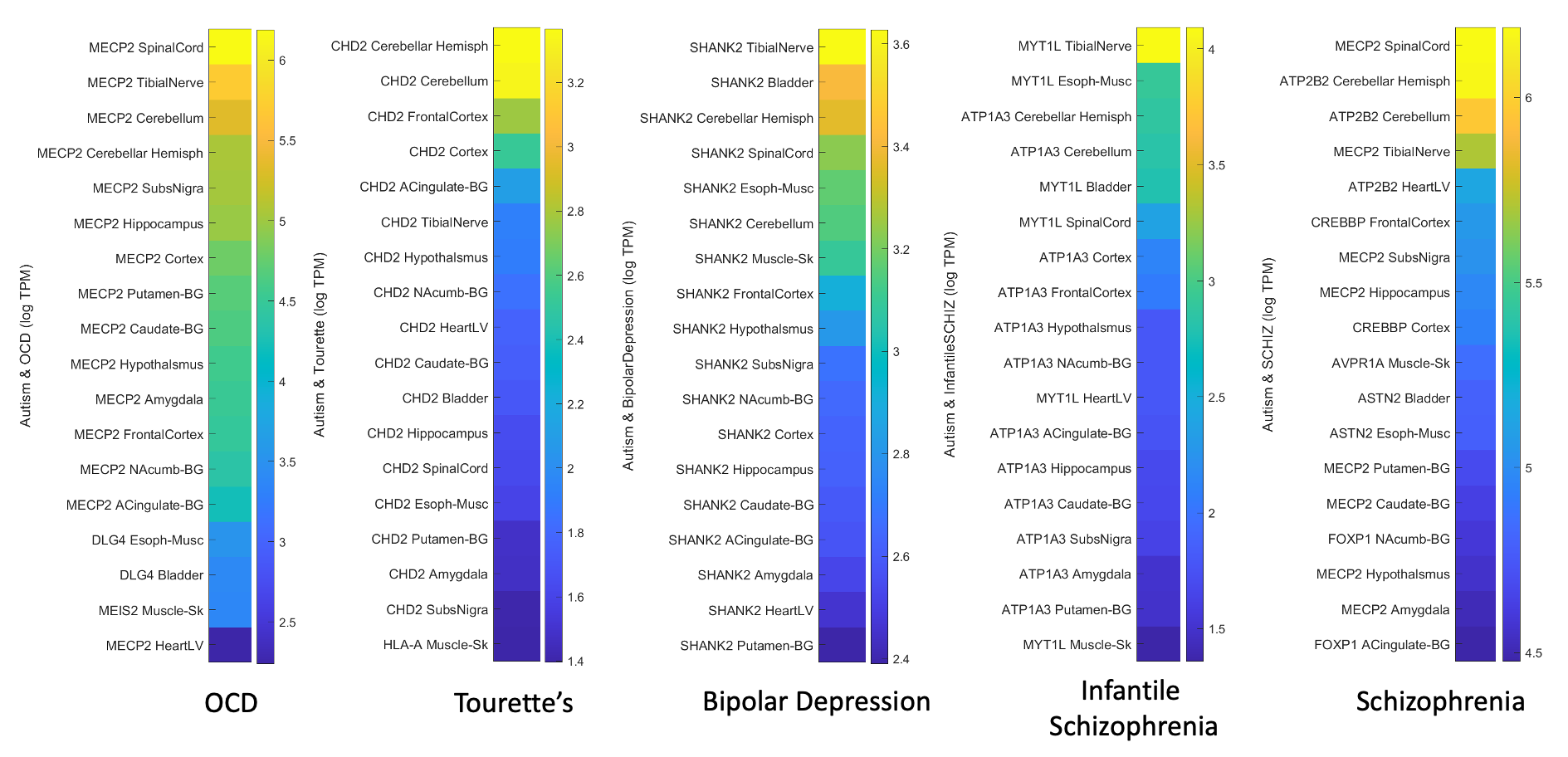

Supplement: Supplementary file 1 [file jpm-11-01119-s001.zip › Supplementary Figure S5.tiff]

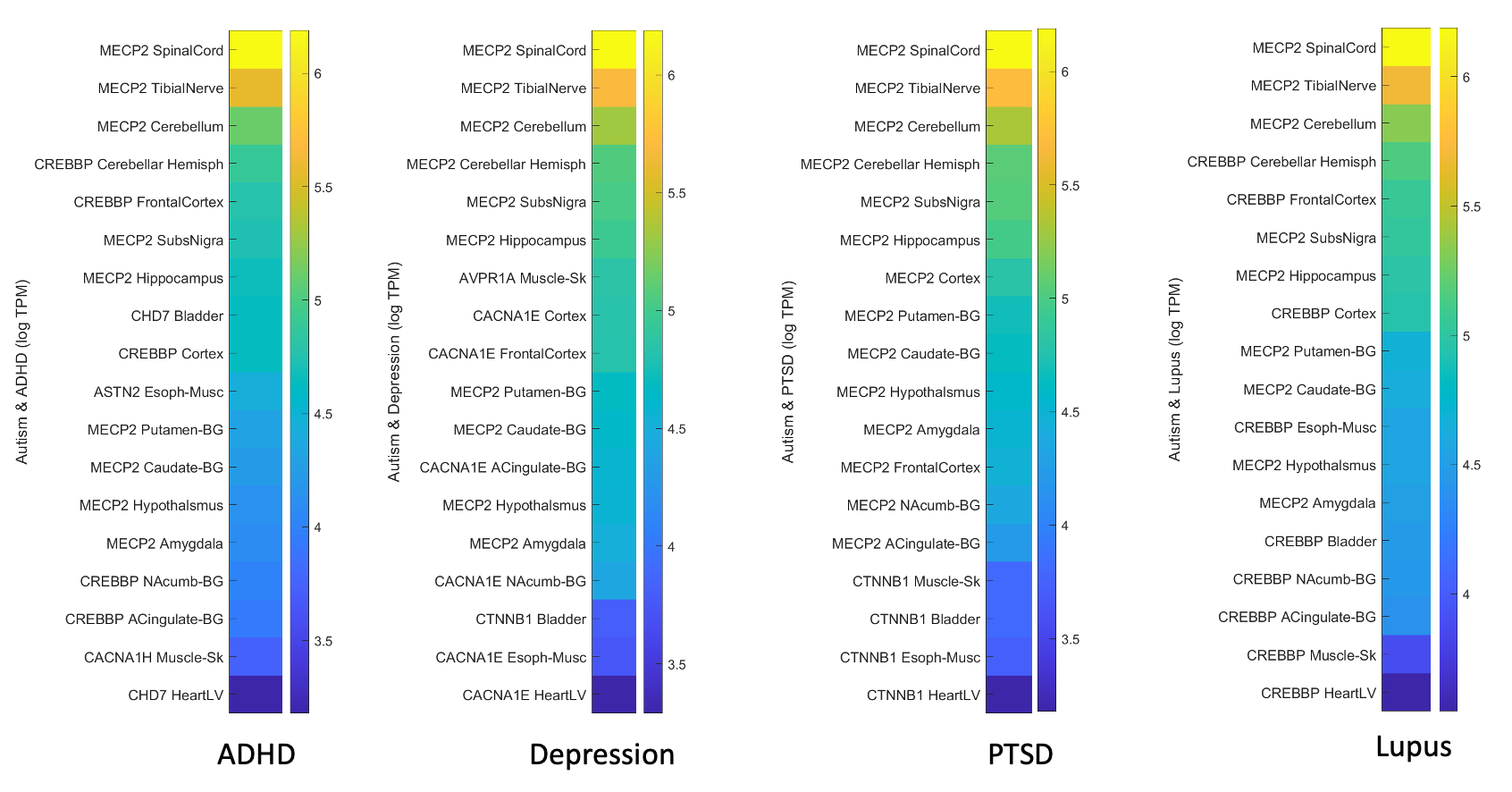

Supplement: Supplementary file 1 [file jpm-11-01119-s001.zip › Supplementary Figure S6.tiff]
